# Medical and Philosophical Intelligence

**Published:** 1813-07

**Authors:** 


### MEDICAL AND PHILOSOPHICAL INTELLIGENCE.

### ROYAL SOCIETY.

On Thursday the 29th of April, the paper by Berzelius and Marcet, on the alcohol of sulphur, was continued. They obtained this substance by subliming sulphur through red-hot charcoal in a porcelaine tube, and receiving the product in water. Thus obtained it was usually of a yellow color, from an excess of sulphur which it contained; but it was reduced to a state of purity by distilling it in a glass retort.

Thus obtained it was a colorless liquid, like water, of a pungent disagreeable taste, and a stronger smell than sulphureted hydrogen gas.

gas. It boiled at the temperature of 110° or 115°; and at the temperature of 53°, when the barometer stood at 30 inches, it was capable of furnishing a vapor which supported a column of mercury 7¼ inches high; so that when mixed with air at the common temperature it increases its bulk one-fourth. It is more volatile than ether, and produced so much cold during its evaporation that mercury was frozen. The alcohol of sulphur may be cooled down to —50° without congealing. It dissolves in alcohol and ether, depositing at the same time its excess of sulphur, if it happen to contain any. It readily dissolves sulphur. Mercury may be boiled in it without any alteration. Potassium, when heated with it, undergoes no change; but when heated in an exhausted retort filled with the vapor of alcohol of sulphur it burns with a red color, a black matter covers its surface, and on admitting water a solution of hepar sulphuris is formed, mixed with charcoal.

To determine if it contained any hydrogen, its vapor was mixed with dry oxygen gas, and detonated by electricity. No water was obtained. Oxymuriatic acid gas was made to pass through it for an hour and a half, and then through water; but no muriatic acid made its appearance, as would have been the case if hydrogen had been present in the alcohol of sulphur. It was made to pass through red-hot muriate of silver; but none of the silver was reduced to the metallic state, as would have been the case if hydrogen had been present. Finally, it was made to pass over several metallic peroxides at a red heat (as red oxide of iron, black oxide of manganese). The oxides were reduced, and converted to sulphurets; but no moisture was deposited in the tube, though surrounded with ice. From all these trials it appears that alcohol of sulphur contains no hydrogen.

On May the 6th, the remainder of the paper by Berzelius and Marcet, on the alcohol of sulphur, was read. The next object was to ascertain the presence of carbon in this oily substance. When burnt in oxygen gas, the residual gas was found to contain sulphurous acid gas. This being removed, some carbonic acid gas remained, which rendered lime-water turbid, and changed pure lime into the carbonate of lime. Both these acid gases being removed, a combustible gas remained, which detonated when mixed with oxygen gas, and was converted into carbonic acid. It was, therefore, carbonic oxide. Alcohol of sulphur being mixed with a caustic ley, with barytes-water, and with lime-water, was slowly decomposed, and a quantity of carbonic acid formed. From these and several other experiments of a similar nature, it follows demonstrably, that the alcohol of sulphur contains carbon. It is in fact a compound of carbon and sulphur, and may therefore with propriety be called *sulphuret of carbon.*

The last object was to determine the proportion of carbon present in this compound. A great variety of methods were tried, such as burning in oxygen gas, decomposition in alkalies, &c. but none of them were found to answer. At last they succeeded, by passing the sulphuret of carbon very slowly through a red-hot tube filled with red oxide of iron. The gaseous products were received over mercury.

The

The red oxide of iron was partly converted into sulphuret. To determine the quantity of sulphur present, it was dissolved in nitro-muriatic acid, and the whole sulphur converted into sulphuric acid. This acid was thrown down by barytes, and its quantity accurately ascertained. The gases over mercury were found to be a mixture of sulphurous acid and carbonic acid. The sulphurous acid, was absorbed by brown oxide of lead, which by that means was converted into sulphate; and the additional weight being ascertained, determined the proportion of sulphurous acid present in the gas. The carbonic acid was absorbed by potash, and its weight determined in the same manner. From these data it was possible to determine the proportion of sulphur and carbon present in the alcohol of sulphur. The result was that it is a compound of

| | |
|---|---|
| Sulphur . . . . . . . | 84·83 |
| Carbon . . . . . . . | 15·17 |
| | 100 |

or of two atoms of sulphur and one of carbon.

On Thursday the 13th of May an appendix to the preceding paper, by Professor Berzelius, was read. It consisted of the four following particulars:—

1. An account of the method employed in determining the proportions of carbon and sulphur in the sulphuret of carbon. The mode was to decompose a given weight of sulphuret of carbon by passing it through red-hot peroxide of iron, and receiving the products over mercury. The sulphuret of iron formed was dissolved, and the sulphur converted into sulphuric acid. The weight of sulphuric acid, of sulphurous acid, and of carbonic acid, formed, was ascertained; and from the known composition of these three substances, the proportion of carbon and sulphur was determined. Two experiments were made. In the first the loss amounted to $\frac{1}{3}$ of a per cent.; in the second to eight thousandth parts.

2. Some observations on the atomic theory. According to Mr. Dalton's theory, sulphuret of carbon is a compound of two atoms of sulphur and one of carbon.[1] Professor Berzelius makes some remarks upon Sir H. Davy's numbers, which he has adopted in his Elements of Chemistry, and shows that they do not answer for the metallic sulphurets with the requisite simplicity. Yet if any sulphuret be treated with an acid so as to convert the metal into an oxide, the quantity of hydrogen disengaged will always indicate exactly the quantity of oxygen in the water decomposed, which would be sufficient exactly to acidify the sulphur. Berzelius thinks that unit ought to be employed to indicate an atom of oxygen, and that the weight of the other atoms should be determined by the proportion in which they combine with oxygen.

3. On the combination of sulphuret of carbon with bases. Berzelius found that sulphuret of carbon combines with ammonia and with lime, the only bases tried. These combinations he calls *carbo-sulphurets.* Carbo-sulphuret of ammonia is formed by putting sulphuret of carbon into a tube, and letting up into it ammoniacal gas as long as it will absorb it. A yellow pulverulent substance is formed, which sublimes unaltered in close vessels, but so deliquescent that it cannot be passed from one vessel to another without absorbing moisture. If it be heated in that state, crystals of hydrosulphuret of ammonia make their appearance. Carbosulphuret of lime is formed by heating some quicklime in a tube, and causing sulphureted carbon to pass through it. The lime becomes incandescent at the time of the combination. On the outside there is formed some sulphuret of lime, which gives it a yellow color. This formation is owing to the action of the air, and is merely superficial.

4. When sulphuret of carbon is left for some weeks in contact with nitromuriatic acid, it is converted into a substance having very much the appearance and physical properties of camphor; being soluble in alcohol and oils, and insoluble in water. This substance Berzelius found to be a triple acid, composed of two atoms of muriatic acid, one atom of sulphurous acid, and one atom of carbonic acid. He proposes to call it *acidum muriatico-sulphuroso-carbonicum.*

On Thursday the 20th of May, a paper by Dr. Reid Clanney, of Sunderland, was read, on a lamp for preventing explosions in coal-mines by the combustion of carbureted hydrogen gas. Dr. Clanney began by giving an historical account of the accidents of this nature which have taken place in the neighbourhood of Sunderland within the last seven years; from which it appears that above 200 workmen have been suddenly killed, and more than 300 women and children left in destitute circumstances by these dreadful explosions. His lamp is extremely simple. It consists of a kind of lantern made air-tight; in which a candle is kept burning. Air is constantly blown into it through water by a pair of bellows to support the combustion, and allowed to escape in the same manner through a valve. By this means no more air can explode than what is within the lantern. Thus no accident can ever happen, and the workmen will be sufficiently warned to make their escape in time.

Two circumstances, connected with this relation, deserve a more accurate investigation than they have yet received. Accidents from fire-damp, in the coal-mines in Scotland, are never known. The accidents are much more frequent in Staffordshire than about Newcastle. Do these differences depend upon the nature of the coal, or on the mode of working the mine?

---

*Linnæan Society.*—May the 4th the remainder of Mr. Anderson's paper on different species of rubus was read. He terminated it with a list of various rare plants which he had observed in Britain, especially in Scotland.

Some quadrupeds from North America were exhibited to the Society by Lord Stanley.

May the 24th, at the Annual General Meeting, the following officers were elected:—

James Edward Smith, M.D. President.
Thomas Marsham, Esq. Treasurer.
Alexander Macleay, Esq. Secretary.
Mr. Richard Taylor, Under Secretary.

The

The five following gentlemen were chosen into the Council:

John Barrow, Esq.
Sir Thomas Gery Cullum, Bart.
Philip Derbishire, Esq.
Mr. James Dickson.
Edward Lord Stanley.

In the room of the five following gentlemen:—

Henry Ellis, Esq.
Thomas Furly Forster, Esq.
Lieut.-Col. Thomas Hardwicke.
Claude Scott, Esq.
George Viscount Valentia.

Since the last General Meeting about four British and three foreign members have died, and 34 new members have been elected; so that the number of fellows at present amounts to 437; the foreign members to 64, and the associates to 40.

---

In a paper published in Philosophical Transactions, Part II. 1812, *On some Combinations of Phosphorus and Sulphur, and on some other Subjects of Chemical Inquiry*, by Sir Humphrey Davy, Knt. LL.D. Sec. R. S. he has determined, 1. that phosphorus combines with two proportions of chlorine. The first of these is a limpid liquid; the second a white sublimate. To the first of these Sir H. Davy has given the name of *phosphorane*. It may be formed by passing the vapour of phosphorus through corrosive sublimate. It is composed of 100 phosphorus united to $333\frac{1}{3}$ of chlorine. It dissolves phosphorus.

The sublimate, called *phosphorana*, is composed of 100 phosphorus united to $333\frac{1}{3} \times 2$ of chlorine, or $666\frac{2}{3}$.

When phosphorane is mixed with water, and slowly evaporated, crystals in the form of four-sided prisms make their appearance. These consist of phosphorous acid combined with water. Phosphorana, treated with water in the same way, forms a thick viscid substance, which consists of phosphoric acid united with water.

2. When these crystals of *hydrophosphorous acid* are heated, they are converted into phosphoric acid, and a peculiar gas escapes, to which Sir Humphrey has given the name of *hydrophosphoric gas*.

Hydrophosphoric gas is not spontaneously combustible; but it explodes when mixed with air, and heated to a temperature rather below 212°. Its specific gravity is 0·87, that of air being 1·00: 100 cubic inches of it, under the ordinary pressure and temperature, weigh 26·53 grains. Its smell is disagreeable, but not so much so as that of phosphureted hydrogen gas: three measures of it require rather more than five measures of oxygen gas for complete combustion. When potassium is heated in it, its bulk is doubled, phosphuret of potassium is formed, and the residual gas is hydrogen. When sulphur is heated in it, the bulk is also doubled, sulphureted hydrogen gas formed, and a compound of sulphur and phosphorus remains. Hence the gas is a compound of 4·5 hydrogen and 22·03 phosphorus, or of 100 hydrogen and 489·56 phosphorus.

 3. When

3. When phosphorus is converted into phosphoric acid, by combustion in oxygen gas, every grain of phosphorus consumes 4½ cubic inches of oxygen. Hence phosphoric acid is composed of 100 phosphorus united to 150·5 oxygen. Phosphorous acid contains just half the oxygen present in phosphoric acid, or it is a compound of 100 phosphorus and 75·25 oxygen.

4. When phosphorus is slowly burnt in the air, the liquid produced is a mixture of phosphoric and phosphorous acids. When phosphorus is burnt in rare air at a moderate heat, the solid acid produces phosphorous acid.

5. The specific gravity of sulphurous acid gas is 2·193, that of air being 1·000, and 100 cubic inches of it under the usual temperature and pressure weigh 66·89 grains. It is composed of equal weights of oxygen and sulphur. When oxygen gas is converted into sulphurous acid gas the bulk is not altered.

6. The specific gravity of sulphureted hydrogen gas is 1·177, that of air being 1·000: 100 cubic inches of it, under the common temperature and pressure, weigh 35·89 grains. It is composed of 100 parts, by weight, of hydrogen, and 1509 of sulphur.

7. Sulphuric acid, free from water, does not appear possible to be formed. Dry sulphurous acid gas and nitrous acid gases have no action on each other.

8. The liquid compound of sulphur and chlorine, which I discovered about eight years ago, is composed of 33 sulphur and 67 chlorine.

9. Water has the property of combining in definite proportions with a great number of bodies, and it has a considerable effect on their properties. In this manner it combines with the earths, alkalies, and most of the metallic oxides.

---

*Method of taking Iron-moulds out of Cotton.*—Cottons of all kinds are apt to receive a dirty yellowish, or orange stain, from iron, which, if allowed to remain, gradually corrodes the cloth and forms a hole. At first these stains are easily removed by means of muriatic acid, or any other diluted acid (except vinegar); but, after they have remained for some time, acids have no effect upon them. It may, therefore, be useful to know a method of removing these moulds in such inveterate cases.

The iron in them is in the state of red oxide; and it appears, from various facts, that the red oxide of iron has a much greater affinity for cotton cloth than the black oxide. The object in view, therefore, should be to bring the iron in the mould to the state of black oxide; after which, muriatic acid will easily remove it. There are two methods of doing this, both of which in the present case answer the purpose completely. The first is to touch the mould with the yellow liquid formed by boiling a mixture of potash and sulphur in water, called hydrogureted sulphuret of potash by chemists. The mould becomes immediately black, and the action of diluted muriatic acid immediately effaces it. The second method is to daub the mould over over with ink so as to make it quite black. After this, muriatic acid takes it out, as in the former case.

---

*Composition of Azote.*—Professor Berzelius has announced, in a letter to a celebrated chemist in London, that he has satisfied himself, by a mode of calculation which he has not explained, that azote is a compound of 44·6 of an unknown inflammable gas, and 55·4 of oxygen gas.

---

*Pure Alumina.*—Mr. Webster lately picked up a very curious mineral upon the beach between Brighthelmstone and Beachy Head. It is a white substance, similar in appearance to a mass of tobacco-pipe-clay; but when examined by Dr. Wollaston was found to consist of pure alumina.

---

*New Patents.*—David Thomas, of Bristol; for a new and improved method of burning animal bones for the purpose of extracting the grease or fat property therefrom, and likewise for extracting the spirituous quality therefrom, and for reducing the remainder, or dry parts of bones, into a substance sufficiently prepared for being ground into ivory black. Dated March 30, 1813.

James Timmins, of Birmingham; for an improved method of making and erecting hot-houses, and all horticultural buildings, and also the making of pine-pits, cucumber-lights, sashes, and church windows. Dated April 7, 1813.

---

Dr. Robert Wall, of Glasgow, has a work in the press on the History, Nature, and Treatment, of Chincough, illustrated by a variety of cases and dissections; to which will be subjoined, an Inquiry into the relative Mortality of the principal Diseases of Children in Glasgow during the last thirty years, and the number who have died at various periods under ten years of age.

---

The expediency of the measure of preventing inoculation for small-pox by an act of the legislature, is again about to be agitated, by a bill presented to the House of Lords, by the Earl of Boringdon, to restrict the propagation of that disease. When this bill was presented, on Monday, the 21st of June, it was observed by Lord Boringdon, "though this country had all the honor of the discovery of Vaccination, yet, from the prejudices existing against it, of all the countries of Europe, this had probably derived the least benefit from the practice. While in other parts of Europe the small-pox had been nearly exterminated, during the last year not fewer than 1200 deaths by small-pox had occurred within the bills of mortality." The object of this bill is not to prohibit small-pox inoculation, but to subject it to such regulations as may prevent the diffusion of the contagion.

*Royal*

*Royal College of Surgeons.*—We whose names are hereunto subscribed, deeply impressed with the many fatal instances of the small-pox, which have lately happened, and which daily occur, in the metropolis, and in various towns of the kingdom: convinced that such events are, in a great degree, consequences of the support and propagation of that disease by inoculation: and, fully satisfied of the safety, and the security, of vaccination: from a consequent sense of duty to the community, do, hereby, engage ourselves, to each other, and to the public, not to inoculate the small-pox, unless, for some special reason, after vaccination; but to pursue, and to the utmost of our power promote, the practice of vaccination.

And, further, we do recommend to all the members of the College, of correspondent opinions and sentiments of duty, to enter into similar engagements.

THOMPSON FORSTER, *Master.*

EVERARD HOME, } *Governors.*

WILLIAM BLIZARD, }

| | | |
|---|---|---|
| JAMES EARLE, | ASSISTANTS. | WILLIAM NORRIS, |
| G. CHANDLER, | | JAMES WARE, |
| CHARLES BLICKE, | | J. A. HAWKINS, |
| T. KEATE, | | F. KNIGHT, |
| J. HEAVISIDE, | | LUDFORD HARVEY, |
| HENRY CLINE, | | WILLIAM LYNN, |
| DAVID DUNDAS, | | JOHN ABERNETHY. |
| JOHN CHARLTON, | | |

---

The project of Lucet for the cure of insanity, now before the public (as far as the projector allows his assumed secret to transpire), under the patronage of the Dukes of Kent, &c. &c. and Dr. Harness, with some other gentlemen not of the medical profession, will be duly noticed as it proceeds. In its present state, the Editors of this Journal can say but little on it; but in its progress it will be noticed to the fullest extent it may deserve.

---

A very singular fraud has been practised for some time past in some of the retail shops in London. Artificial pepper-corns, both white and black, are mixed with real pepper-corns, and this fraudulent mixture sold as genuine pepper. The mode of detecting the cheat is easy. Throw a handful of the suspected pepper-corns into water: the artificial corns fall to powder, or are partially dissolved; while the true pepper-corns remain whole. These fraudulent pepper-corns are made of peasemeal. The fraud should be publicly known, because such a mixture, if used instead of real pepper, may prove, in many cases of household economy, exceedingly prejudicial to those who ignorantly make use of it.

